# Role of Inflammation in the Pathogenesis of Diverticular Disease

**DOI:** 10.1155/2019/8328490

**Published:** 2019-03-14

**Authors:** Antonio Tursi, Walter Elisei

**Affiliations:** ^1^Gastroenterology Service, ASL BAT, Andria, Italy; ^2^Gastroenterology Unit, ASL Roma 6, Albano Laziale, Roma, Italy

## Abstract

Diverticulosis of the colon is the most common condition in Western societies and it is the most common anatomic alteration of the human colon. Recurrent abdominal pain is experienced by about 20% of patients with diverticulosis, but the pathophysiologic mechanisms of its occurrence are not completely understood. In the last years, several fine papers have showed clearly the role of low-grade inflammation both in the occurrence of symptoms in people having diverticulosis, both in symptom persistence following acute diverticulitis, even if the evidence available is not so strong. We do not know yet what the trigger of this low-grade inflammation occurrence is. However, some preliminary evidence found colonic dysbiosis linked to low-grade inflammation and therefore to symptom occurrence in those patients. The aim of this paper is to summarize current evidences about the role of inflammation in symptom occurrence in symptomatic uncomplicated diverticular disease and in symptom persistence after an episode of acute diverticulitis.

## 1. Introduction

Diverticulosis of the colon is the most frequent anatomic alteration that can be detected during colonic exploration, and its incidence is increasing worldwide. It is a common finding especially in developed countries: by the age of 85, the large majority of the population living in developed countries will have developed colonic diverticula [1]. Recent epidemiology studies found different prevalence in European countries according to socioeconomic status: countries with a lower socioeconomic status report a 5.3% frequency of diverticulosis in patients aged 30–39, 8.7% in those aged 40–49, 19.4% between 50 and 59, and up to 29.6% in subjects over the seventh decade, while maximal incidences were found in patients aged 70–79 and above 80 of 40.2% and 57.9%, respectively [2]. Although diverticulosis prevalence in Africa and Asia remains low, more recent epidemiology studies found its prevalence increasing also in the developing world, probably because of changes in lifestyle [2].

Most people with colonic diverticulosis remain asymptomatic. Just about one-fourth to one-fifth of those people will develop symptoms without complications, experiencing the so-called “Symptomatic Uncomplicated Diverticular Disease” (SUDD), and about 20-25% of them will ultimately develop diverticulitis (uncomplicated or complicated) [3–6].

Although advancing age is obviously associated with diverticulosis, the occurrence of symptoms is probably not only linked to age but also to the prolonged time course, during which the colonic wall is exposed and makes the colon more susceptible to other pathogenetic factors.

The pathogenesis of the disease is poorly understood and several etiological factors may play a role in its onset. In this paper, we focused on the roles of inflammation as part of the pathophysiology of diverticular disease.

## 2. Pathophysiology of Diverticulosis

Diverticulosis is characterized by the presence of sac-like protrusions of mucosa and submucosa, called diverticula, which occur in the weakest point of the muscle layer. This occurs due to age-related degeneration of the mucosal wall and segmental increases in colonic pressure resulting in bulging at points of weakness, typically at the insertion of the *vasa recta* [3].

Although the underlying pathological mechanisms that cause the formation of colonic diverticula remain unclear, itis likely to be the result of complex interactions between genetic factors, age, diet, changes in colonic structure and colonic motility, and therefore colonic microbial imbalance [3].

Inflammation probably does not play any role in the occurrence of colonic diverticulosis. Two European papers, published at the beginning of this decade, found that no signs of histological inflammation were found in people having colonic diverticulosis [7, 8]. These data have been recently confirmed by a study conducted in the United States. Peery et al. analyzed data about colonic mucosal biopsy specimens from people having diverticulosis. Assessing levels of interleukin (IL) 6 (IL6), IL10, tumor necrosis factor (TNF), and numbers of immune cells (CD4+, CD8+, CD27+, and mast cell tryptase), they found that there was no association between diverticulosis and TNF (odds ratio (OR), 0.85; 95% Confidence Interval (CI), 0.63-1.16) and no association with CD4+ cells (OR, 1.18; 95% CI, 0.87-1.60), CD8+ cells (OR, 0.97; 95% CI, 0.71-1.32), or CD 57+ cells (OR, 0.80; 95% CI, 0.59-1.09). Compared with controls without diverticulosis, biopsy specimens from people having diverticulosis were less likely to express the inflammatory cytokine IL 6 (OR, 0.59; 95% CI, 0.36-0.96). Moreover, there was no association between diverticulosis and irritable bowel syndrome (IBS) (OR, 0.53; 95% CI, 0.26-1.05) [9]. Theoretically, we cannot exclude that inflammation could play a role in the pathogenesis of diverticulosis, but this role is just marginal. At present, inflammation does not seem to show a significant role in the pathogenesis of diverticulosis.

## 3. Inflammation in the Pathogenesis of Symptoms and Clinical Setting

As stated, just one-fourth to one-fifth of patients with diverticulosis develop symptoms, the so-called Symptomatic Uncomplicated Diverticular Disease (SUDD). The pathogenetic mechanisms that lead to the occurrence of symptoms are still under debate, probably because there is still no consensus about the definition forms of the disease.

SUDD is characterized by nonspecific attacks of abdominal pain without macroscopic evidence of an inflammatory process, with abdominal pain, bloating, and changes in bowel habits that may resemble IBS. As a consequence, several authors still talk about “IBS with diverticulosis.” For example, Jung et al. found that IBS occurs 4.7-fold more likely in patients after an episode of acute diverticulitis than controls [10], and more recently, Järbrink-Sehgal et al. found that diverticulosis was associated with diarrhea-predominant IBS (OR, 9.55; 95% CI, 1.08-84.08; *p* = 0.04) [11].

It is likely that those patients are suffering from SUDD than from “IBS with diverticulosis,” even when we describe symptoms' occurrence/persistence following an episode of acute diverticulitis. This is because IBS and SUDD are not the same clinical entity, as showed by clinical data currently available. IBS and SUDD do not share the same epidemiology [12] but only part of clinical features, and patients with SUDD do not generally fulfil IBS criteria [13, 14]. Moreover, the characteristics of the abdominal pain (left lower-abdominal pain lasting for more than 24 h characterizes SUDD, diffuse and short-lived abdominal pain characterizes IBS) seem likely the most effective clinical tool in discriminating between patients with SUDD with those having IBS [13, 14].

## 4. Inflammation and Pathogenesis of Acute Diverticulitis

As it is well known, acute diverticulitis (AD) shows significant sign of inflammation [3]. However, its real pathogenesis is still unclear. Inflammation could play a pathogenetic role in explaining some finding, in particular in acute uncomplicated diverticulitis (AUD), but not at all. For example, we know that the majority of patients having acute complicated diverticulitis (ACD) are at the first appearance of the disease, without any significant symptom before complication [15]. Recently, a review article underlines two other mechanisms explaining the AD occurrence, the “traumatic” and the “ischemic” hypotheses [16]. The “traumatic” mechanism takes into account the damage to the mucosa due to fecalith impaction, which occurs in the large diverticula. However, not uncommonly, acute diverticulitis (AD) develops in young patients with only few and small diverticula, where fecalith trapping is very unlikely. The “ischemic” mechanisms may be caused by the compression of vascular structures in the neck of the diverticular task, as a result of prolonged and/or recurrent contractile spikes related to neuromuscular alterations in the diverticular tract. Clearly, the “traumatic” and “ischemic” mechanisms of acute diverticulitis are not mutually exclusive and may act in different patients.

Also, inflammation may play a role in the pathogenesis of the disease. Dai et al. showed a different expression of prostaglandin E_2_-related enzyme in patients with AD. In particular, they observed a downregulation of cyclooxygenase-2/PGE_2_ and upregulation of 15-PGDH in the human colonic mucosa that may be predisposing factors for the development of acute DD [17]. Based on this finding, authors hypothesized that the change of COX-2/15-PGDH and the resulting decrease of PGE_2_ may predispose to AD occurrence. Moreover, we found that AD and Crohn's disease (CD) have a similar pattern of expression of basic fibroblast growth factor (bFGF), syndecan 1 (SD1), and tumor necrosis factor *α* (TNF *α*), supporting the hypothesis that inflammation may also activate the fibrosis process [18].

About the role of TNF*α*, its overexpression in AD as well as CD is not regulated by the same gene superfamily 15 (TNFSF15), which is an immunoregulatory, antiangiogenic gene [19, 20]. A recent study found that not all TNFSF15 superfamilies are shared among these two diseases. In particular, the CD GAGGA haplotype was significantly associated with diverticulitis (*p* = 0.03) in all DD versus all control comparison. A second haplotype, rs6478108 (A), rs6478109 (G), rs7869487 (A), and rs4263839 (G), was also associated with DD in that cohort (*p* = 0.025). A third haplotype, rs6478108 (A), rs6478109 (G), rs7848647 (G), rs7869487 (A), and rs4263839 (G), was demonstrated in the DD < 55 years versus control > 55 years' comparison (*p* = 0.045). This study demonstrates clearly that distinct but overlapping TNFSF15 haplotypes can be found in diverticulitis patients versus healthy controls when compared with the known CD haplotype suggesting similar but distinct genetic predispositions [21]. This study strengthens therefore the role for a genetic predisposition to diverticulitis that involves the TNFSF15 immunoregulatory gene [22], triggering the occurrence of inflammation as a consequence.

## 5. Inflammation and Symptoms' Persistence following Acute Diverticulitis

We know that up to 20% of patients complain of persistent abdominal pain after surgical treatment of diverticulitis [23], and the quality of life of those patients is significantly worse [24]. Persistence of symptoms following an episode of AD has been linked to several factors, ranging from significant attenuation in serotonin-transporter expression to persistence of low-grade inflammation [25, 26]. Persistent low-grade inflammation of the colon may also explain clinical symptom persistence following AD, even if surgically treated. In fact, these findings have been recently confirmed by Lahat. Using reverse transcription polymerase chain reaction-determining cytokine levels, authors found that TNF*α*, interleukin- (IL-) 6, and IL-1*β* levels were significantly higher and histology was significantly worse in patients having symptom persistence following severe AUD (*p* < 0.05 for all comparisons) [27].

## 6. Inflammation and Risk of Acute Diverticulitis Recurrence

Persistent low-grade inflammation following an episode of AD may also explain the recurrence of AD. At the end of a 24-month follow-up period, we found that endoscopic inflammation was still detected in 27.67% of patients and active histological inflammation in 36.6% of patients following an episode of AD. Only the detection of endoscopic and of histological inflammation during the follow-up was a predictor of diverticulitis recurrence (*p* = 0.0004) [26].

Inflammation persistence may be therefore a risk factor for both symptoms' persistence and disease recurrence. Despite this, a recent review found mesalazine, an anti-inflammatory drug widely used in the treatment of Inflammatory Bowel Diseases, not superior to placebo in preventing diverticulitis recurrence [28]. It is probably due to the heterogeneity of the current trials and the heterogeneity of the patients enrolled in those trials [29]. Although it is advisable that further randomized, double-blinded, placebo-controlled trials of rigorous design have to be performed to specify the effects of mesalazine in the management of diverticulitis, we mean that this is the best evidence that could ever be obtained on this topic, since mesalazine is now out of patent and it is unlikely that any large RCT could ever be sponsored.

## 7. Inflammation and Pathogenesis of Symptomatic Uncomplicated Diverticular Disease

As stated, diverticulosis is merely the presence of colonic diverticula without symptoms or macroscopic/microscopic signs of inflammation. In this way, no signs of inflammation should be detected, both at endoscopic and also at histological assessment.

A different scenario may be observed when we analyze patients with SUDD. In those patients, low-grade inflammation seems to really play a significant role in determining symptom occurrence and complication. The current evidences are as follows.

### 7.1. Microscopic Inflammation

SUDD shows a significant microscopic inflammatory infiltrate. More than ten years ago, Narayan and Floch found a significant inflammatory infiltrate in SUDD patients compared with healthy controls [30]. We assessed both neutrophilic and lymphocytic infiltrate in SUDD, AUD, and healthy controls (HC). While neutrophilic inflammatory infiltrate was found only in AUD, the mean lymphocytic cell density was significantly higher in SUDD than in asymptomatic diverticulosis and HC (*p* < 0.02).

### 7.2. Enhanced Expression of TNF*α*

TNF-*α*, a 17 kDa polypeptide produced by macrophages, lymphocytes, and natural killer cells, has been shown to play a major role in the inflammatory process, with high levels being found both in Inflammatory Bowel Disease (IBD) and in rheumatoid arthritis patients [31–33]. Hypothesizing a role of low-grade inflammation in the occurrence of SUDD, TNF*α* was assessed in those patients yet.

Assessing its mucosal expression on biopsy samples by reverse transcription polymerase chain reaction, we found that TNF*α* expression was significantly higher not only in AUD than in HC (*p* = 0.0007) but also in SUDD than in HC (*p* = 0.0007) and in asymptomatic diverticulosis (*p* = 0.0001). Moreover, TNF*α* expression in AUD did not differ significantly from that of UC (*p* = 0.0678) and SCAD (*p* = 0.0610). Finally, it was significantly higher in UC, SCAD, and AUD than in SUDD (*p* = 0.0007, *p* = 0.0001, and *p* = 0.0179, respectively) [34]. The same results were drawn by Humes et al., who found that TNF*α* expression was significantly higher in SUDD than in asymptomatic diverticulosis (*p* = 0.04) [8].

### 7.3. Paradoxical Expression of IL-10

IL-10 is an important anti-inflammatory mediator that suppresses the production of proinflammatory cytokines [35]. Surprisingly, IL-10 expression in SUDD seems to have an opposite behaviour than expected. Turco et al. found that inducible nitric oxide synthase (iNOS) expression was significantly increased in SUDD and SUDD following acute diverticulitis (SUDD-AD) patients compared to controls (*p* < 0.05). Moreover, they found a slight increase of IL-10 release in SUDD and a significant IL-10 release in SUDD-AD than HC (*p* < 0.05) [36]. We recently confirmed these results. Using an Enzyme-Linked Immunosorbent Assay (ELISA) analysis on fecal samples, we found an increased release of IL-10 from HC up to asymptomatic diverticulosis and SUDD patients. In particular, mean IL-10 expression was 3.32 (95% CI 0.43-7.91) pg/g in HC, 3.42 (95% CI 1.87-5.10) pg/g in asymptomatic diverticulosis, and 4.24 (95% CI 1.90-7.53) pg/g in SUDD. However, no significant difference was found among the 3 study groups (*p* = 0.326) [37].

Thus, we can speculate that IL-10 expression in SUDD may be an attempt of the immune system to control a low-grade inflammation. This hypothesis seems to be confirmed by Dai et al., who found an inverse expression of prostaglandin E_2_ in colonic mucosa between acute diverticulitis and IBD [17]. We know that prostaglandin E_2_ is the dominant prostaglandin in the colon and it is associated with colonic inflammation, but we also know that it has a protective mechanism in the mucosa of the gastrointestinal tract. Lower prostaglandin E_2_ level may decrease the normal protection of the mucosa, which is made more susceptible to luminal insults, thus creating a permissive environment for the development of acute diverticulitis.

### 7.4. Fecal Calprotectin Overexpression

Fecal calprotectin (FC) is a cytoplasmic antimicrobial compound prominent in granulocytes, monocytes, and macrophages, and it accounts for approximately 60% of the total cytosolic protein. It is released from cells during cell activation or death, and it is stable in feces for several days after excretion [38]. This has been shown to be a sensitive marker of activity both in CD and in Ulcerative Colitis (UC); it is the stronger inflammatory marker predictor of relapse in those people as well [39].

After a preliminary report describing an increase of FC expression in patients with “symptomatic diverticular disease” (no more information were provided by authors) [40], we performed a case control study assessing FC in asymptomatic diverticulosis, SUDD, AUD, IBS, and HC patients. FC was not increased in HC and IBS patients; no difference was found between asymptomatic diverticulosis, HC, and IBS patients as well (*p* = n.s.). Higher FC values were found in AUD (*p* < 0.0005) and SUDD (*p* < 0.005) than in HC and IBS patients. Moreover, FC values correlated significantly with inflammatory infiltrate (*p* < 0.0005) and decreased to normal values after treatment both in AUD (*p* < 0.0005) and in SUDD (*p* < 0.005) [41]. Although this study is limited by using a semiquantitative method, which requires careful evaluation in patients with small or aqueous feces [42] and does not provide a clear cut-off, it suggests that FC may be a useful tool in detecting inflammation in the colon harboring diverticula, distinguishing symptoms coming from diverticular inflammation than that coming from IBS overlapping diverticulosis.

### 7.5. Significant Benefit When Using Mesalazine

Several clinical trials in SUDD patients found 5-aminosalicilic acid (alone or in combination) [43–45] effective in reducing SUDD symptoms and in primary prevention of acute diverticulitis, further supporting the possibility that chronic inflammation occurrence during the course of diverticular disease plays a role in its pathogenesis.

Some studies tried to show that these are flaw evidences, since patients having diverticulosis and chronic abdominal symptoms do not show any evidence of inflammation [9]. However, the definition of “abdominal chronic symptoms in diverticulosis” does not fulfil the current definition of SUDD [46]. On the contrary, patients fulfilling the correct definition of SUDD show all the reported evidences on the role of inflammation in the pathogenesis of SUDD [47].

## 8. Relationship between Low-Grade Inflammation and Other Mechanisms Leading to Symptoms' Occurrence

Low-grade inflammation may also interact with other pathogenetic mechanisms to lead symptom occurrence.

For example, visceral hypersensitivity has been suggested as a possible mechanism involved in causing symptoms in those patients [48, 49]. Humes et al. found that SUDD patients had greater expression of neurokinin 1 (NK1) than the asymptomatic patients with diverticulosis (*p* = 0.01); a higher but not significant expression of galanin 1 receptor (GALR1) in SUDD than asymptomatic patients was found [8] as well. The increase in expression of the inflammatory cytokines IL-6 and TNF*α*, and an upregulation of the neuropeptide receptor NK1, suggests that SUDD patients may exhibit visceral hypersensitivity due to peripheral sensitization with both inflammatory and neurochemical factors playing a role.

Recent studies demonstrated that DD is associated with peculiar alterations of the enteric nervous system (ENS), encompassing myenteric oligoneuronal hypoganglionosis, decreased intramuscular nerve fibers, and altered neurochemical coding [40–53]. Analyzing the expression of the glial markers S100*β*, Cossais et al. found very recently that S100*β* expression was increased in the submucosal and myenteric plexus of patients with DD compared with that in controls, whereas the expression of other glial factors remained unchanged. Moreover, this S100*β* overexpression was correlated to CD3+ lymphocytic infiltrates in patients with DD, whereas no correlation was observed in controls [54].

### 8.1. Why Inflammation Occurs in Diverticular Disease: The Role of Colonic Microbial Imbalance

It has been hypothesized that microbial imbalance, which is known as dysbiosis, may be the trigger of inflammation (and therefore symptoms) in people having diverticulosis [55]. Daniels et al. found that patients at the first episode of acute diverticulitis have a higher diversity of *Proteobacteria* than controls [56], and Schieffer et al. found that *Microbacteriaceae* were the most significant species associated with the disease [57]. Microbiota imbalance was found also in SUDD, sometimes significantly differing from that reported occurring in Inflammatory Bowel Diseases (IBD). For example, we know that *Akkermansia muciniphila*, a mucolytic bacteria living in the mucus barrier that degrades mucin for utilization as a carbon and nitrogen energy source [58] and producing short-chain fatty acids to maintain intestinal homeostasis [59], is significantly decreased in IBD [60]. The same occurs when we assess the relative abundance in IBD of another mucolytic bacteria, *Roseburia hominis* [61]. In SUDD, it seems the opposite: *A. muciniphila* and *R. hominis* have a significant abundance in SUDD [62–65], and their decrease by therapy is associated to a significant improvement of symptoms [62–65].

## 9. Conclusion

Diverticulosis is a common condition that sometimes becomes symptomatic and may lead to severe complications.

**Figure 1:**
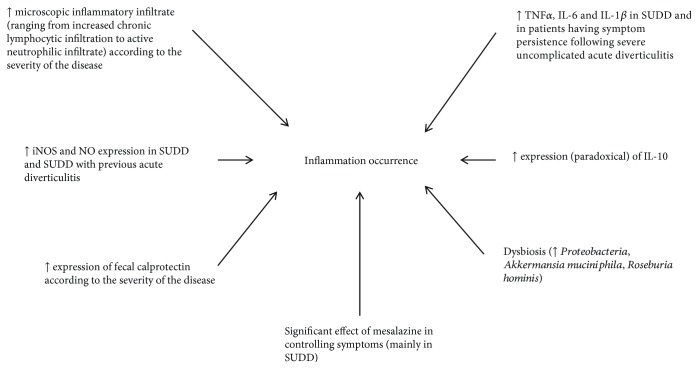
Current evidences on the role of inflammation in the pathogenesis of diverticular disease.

The pathophysiologic hypotheses behind symptom occurrence in those patients have changed in the last years. Although most of the mechanisms leading to its occurrence remain to be elucidated, the role of low-grade inflammation seems to be a proven fact (Figure 1 reassumes the main concepts discussed in the manuscript).

Why inflammation occurs in only a minority of people having diverticulosis is still under debate, and it has to be further elucidated.
